# The Role of Ferrous Ion in the Effect of the Gadolinium-Based Contrast Agents (GBCA) on the Purkinje Cells Arborization: An In Vitro Study

**DOI:** 10.3390/diagnostics11122310

**Published:** 2021-12-08

**Authors:** Achmad Adhipatria Perayabangsa Kartamihardja, Winda Ariyani, Hirofumi Hanaoka, Ayako Taketomi-Takahashi, Noriyuki Koibuchi, Yoshito Tsushima

**Affiliations:** 1 Department of Diagnostic Radiology and Nuclear Medicine, Gunma University Graduate School of Medicine, Maebashi 371-8511, Japan; akartamihardja@gunma-u.ac.jp (A.A.P.K.); ayakorad@gunma-u.ac.jp (A.T.-T.); 2 Department of Nuclear Medicine and Molecular Imaging, Universitas Padjajaran, Bandung 40161, Indonesia; 3 Department of Integrative Physiology, Gunma University Graduate School of Medicine, Maebashi 371-8511, Japan; winda@gunma-u.ac.jp (W.A.); nkoibuch@gunma-u.ac.jp (N.K.); 4 Department of Bioimaging and Information Analysis, Gunma University Graduate School of Medicine, Maebashi 371-8511, Japan; hanaokah@hirakata.kmu.ac.jp; 5 Division of Integrated Oncology Research, Gunma Initiative for Advanced Research, Gunma University Graduate School of Medicine, Maebashi 371-8511, Japan

**Keywords:** gadolinium (Gd), gadolinium-based contrast agent (GBCA), Gd toxicity, transmetallation, Purkinje cells, neuron morphogenesis

## Abstract

Gadolinium deposition in the brain has been observed in areas rich in iron, such as the dentate nucleus of the cerebellum. We investigated the role of Fe^2+^ in the effect of gadolinium-based contrast agents (GBCA) on thyroid hormone-mediated Purkinje cell dendritogenesis in a cerebellar primary culture. The study comprises the control group, Fe^2+^ group, GBCA groups (gadopentetate group or gadobutrol group), and GBCA+Fe^2+^ groups. Immunocytochemistry was performed with an anti-calbindin-28K (anti-CaBP28k) antibody, and the nucleus was stained with 4′,6-diamidino-2-phenylindole (DAPI). The number of Purkinje cells and their arborization were evaluated with an analysis of variance with a post-hoc test. The number of Purkinje cells was similar to the control groups among all treated groups. There were no significant differences in dendrite arborization between the Fe^2+^ group and the control groups. The dendrite arborization was augmented in the gadopentetate and the gadobutrol groups when compared to the control group (*p* < 0.01, respectively). Fe^2+^ significantly increased the effect of gadopentetate on dendrite arborization (*p* < 0.01) but did not increase the effect of gadobutrol. These findings suggested that the chelate thermodynamic stability and Fe^2+^ may play important roles in attenuating the effect of GBCAs on the thyroid hormone-mediated dendritogenesis of Purkinje cells in in vitro settings.

## 1. Introduction

Chelated gadolinium (Gd)-based contrast agents (GBCA) have an excellent safety profile for clinical magnetic resonance imaging (MRI) [1]. Given Gd’s toxic nature, chelation is critical for its safety profile [2]. Although chelation greatly improves the safety profile of GBCA [3], previous studies showed that Gd retention may occur in various organs, including in the brain tissue of healthy humans [4,5,6]. Although Gd from GBCAs may be deposited in various areas of the brain, the effect of Gd retention in the brain has yet to be fully explained.

Gd retention in the brain may pose a threat to the neurons themselves. Previous studies showed that Gd^3+^ may damage cortical neurons through the oxidative stress pathway [7,8]. The highest Gd retention was observed in areas of the brain rich in iron, such as the dentate nucleus of the cerebellum [9]. The thermodynamic stability of chelated iron is higher than chelated gadolinium [10]. Therefore, it may be involved in the Gd transmetalation phenomenon [11] and may explain why an area with a high iron concentration has a higher Gd retention. In biological media, endogenous ions like iron and zinc may form very stable complexes with chelate ligands, and even highly stable Gd-chelates will release a small amount of free Gd [3]. Concurrently, Gd has a high affinity for phosphate, citrate, and carbonate ions, and will bind to proteins like serum albumin [3,12]. Thus, excess iron would favor the dissociation of Gd from its chelate.

Occasionally, radiologists may use Gd contrast-enhanced MRI (CE-MRI) in patients with iron overload, including pediatric patients [13]. CE-MRI is also used during pregnancy, during which iron supplementation is frequent [14]. Therefore, contrast-enhanced MRI in patients with high iron concentrations may need careful consideration because of its potential neurotoxicity.

Thyroid hormone (TH) plays a critical role in normal mammalian brain development and functional maintenance [15]. A previous study showed that exposure to gadodiamide or gadoterate meglumine altered TH receptor (TR) action and TH-induced cerebellar Purkinje cell morphogenesis [16]. In addition, iron, as the strongest candidate for inducing the transmetalation of GBCA, might affect TR action and TH-responsive genes [17,18,19,20].

The aim of this study was to investigate the thyroid hormone-mediated morphological alteration of cerebellar Purkinje cells in vitro after their exposure to linear or macrocyclic chelate GBCAs in the presence of Fe^2+^.

## 2. Materials and Methods

### 2.1. GBCAs and Ferrous Iron

Gadopentetate dimeglumine (Magnevist^®^, linear GBCA) and Gadobutrol (Gadovist^®^, macrocyclic GBCA) were purchased from Bayer Yakuhin Ltd., Osaka, Japan. The GBCAs were diluted in the culture medium to treatment doses of 1 nM, 10 nM, or 100 nM. Ferrous sulfate (Fe^2+^; MW, 278.01) was purchased from Fujifilm Wako Pure Chemical Industries, Ltd. (Osaka, Japan). We dissolved Fe^2+^ powder in the culture medium, filtered it with a 0.22 µm membrane to a stock concentration of 1 µM, and stored it at −20 °C until use. The treatment dose of Fe^2+^ was 10 nM.

The experiments were replicated three times. Each experiment consisted of 6 major groups; a control group, an Fe^2+^ group (10 nM), a gadopentetate group (1 nM, 10 nM, and 100 nM), a gadobutrol group (1 nM, 10 nM, and 100 nM), a gadopentetate-Fe^2+^ group, and a gadobutrol-Fe^2+^ group. In the gadopentete-Fe^2+^ group, 10 nM Fe^2+^ was incubated with 1 nM, 10 nM, or 100 nM gadopentetate. In the gadobutrol-Fe^2+^ group, 10 nM Fe^2+^ was incubated with 1 nM, 10 nM, or 100 nM gadobutrol.

### 2.2. Primary Cerebellar Culture

The study was conducted according to the guidelines of the Declaration of Helsinki and approved by the Institutional Review Board of Gunma University (Experiment protocol no. 20-037, 06/08/2020). Pairs of C57BL/6 mice (Japan SLC, Inc., Hamamatsu, Japan) were bred in the local institution. The procedure minimized the number of animals used and their suffering under the local animal care and experimentation committee guidelines. A total of thirteen independently randomized litters were used in this study. The cerebellum was isolated from decapitated pups on the first day of birth (P0) based on previously established culture methods [21]. In brief, the cerebellum was collected under a dissecting microscope in a culture hood. Freshly isolated cerebellum was digested with 0.2 U/mL of papain (Worthington, Lakewood, NJ, USA) in phosphate-buffered saline (PBS) containing 0.2 mg/mL l-cysteine, 0.2 mg/mL, 5 mg/mL glucose (Sigma-Aldrich, St. Louis, MO, USA) bovine serum albumin (Intergen Company, Purchase, NY, USA), and 0.02 mg/mL DNase I (400–600 U/mg; Sigma-Aldrich). The procedure was done at 36.5 °C in a water bath equipped with a shaker for 25 min. Following centrifugation, the dissociated cells were suspended in Ham’s F12-Dulbecco’s modified essential medium (DMEM/F12, serum free; (Sigma-Aldrich) and plated on poly-L-lysine coated chamber slides (Lab-Tek 8 mm diameter wells, Nalge Nunc International, Rochester, NY, USA) at a density of 3 × 10^5^ cells per well. Twenty-four hours later, the medium was changed with F12-DMEM medium supplemented with 1% antibiotics, 10% fetal bovine serum (FBS), and 1 nM thyroxine T4 (Sigma-Aldrich). GBCAs and 10 nM Fe^2+^ were also added to the medium of the relevant groups at this time. Part of the medium (200 µL) was replaced with fresh medium every 3 days, and the cells were cultured in an incubator (37 °C, 5% CO_2_) for 17 days.

### 2.3. Immunocytochemistry for Calbindin to Analyze Purkinje Cell Morphology

The immunocytochemistry of the cultured cells was performed as previously described [21]. In brief, the cells were fixed by 4% paraformaldehyde, followed by cell permeation with 0.1% nonionic surfactant (Triton™ X-100; Sigma-Aldrich). Immunochemical staining was performed with a 1:200 mouse monoclonal anti-calbindin-28K (anti-CaBP28k) primary antibody and a donkey anti-mouse IgG (H + L) secondary antibody, Alexa Fluor^®^ 488 conjugate (1:200; Thermo Fisher Scientific Inc., Waltham, MA, USA). Cell nuclei were stained with 4′,6-diamidino-2-phenylindole (DAPI). Ten images of Purkinje cells were randomly captured from each well (per experiment) with the laser confocal scanning microscope ZEISS LSM 880 (Carl Zeiss Microscopy GmbH, Jena, Germany).

ImageJ software (NIH) was used to quantify the relative dendritic area (dendrite arborization) in the area covered by the dendritic tree, which was determined by tracing the outline of the cell and its dendritic branches. The numbers of Purkinje cells (CaBP28k-positive cells) per well (1 cm^2^) were counted manually using a schematic grid from the top right corner to the lower left corner of the well.

### 2.4. Statistical Analysis

All data were expressed as means ± standard deviation (SD). An analysis of variance was performed to analyze the treatment effect of GBCAs and their interaction with Fe^2+^ (η2). A post-hoc multiple comparison to determine which category was significantly different was done by a Tukey honest significant difference (HSD) test. SPSS software (version 23; IBM-SPSS, Inc., Chicago, IL, USA) was used for data analyses. All *p* values of less than 0.05 were considered statistically significant.

## 3. Results

### 3.1. Purkinje Cell Number

**Figure 1:**
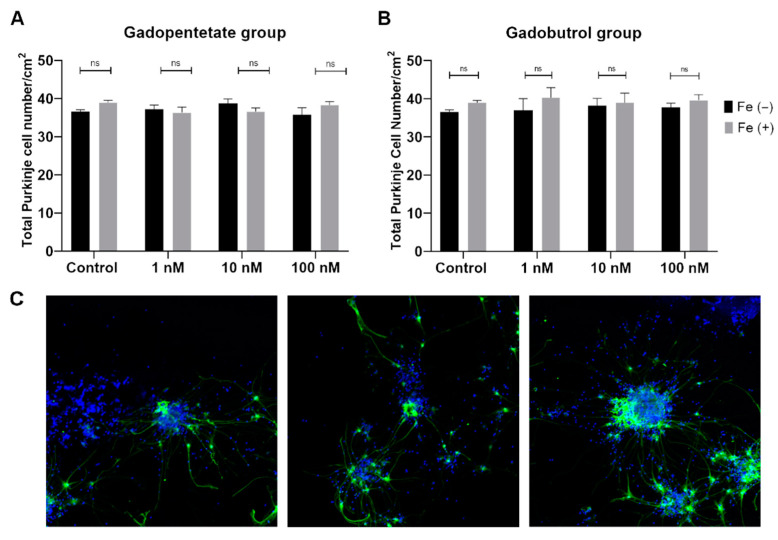
Total Purkinje cell number of the (**A**) gadopentetate group and (**B**) gadobutrol group. There were no differences in cell number among all treated groups when compared to the control group. (**C**) Representative images of the cells at low magnification (10×). ns: not significant.

The Purkinje cell numbers per well were similar between the Fe^2+^ group and the control group. There were also no differences among the gadopentetate and gadopentetate-Fe^2+^ groups (Figure 1A), and among the gadobutrol and gadobutrol-Fe^2+^ groups (Figure 1B). The representative images for cell numbers quantification was described in Figure 1C.

### 3.2. Dendrite Arborization of the Purkinje Cells

**Figure 2:**
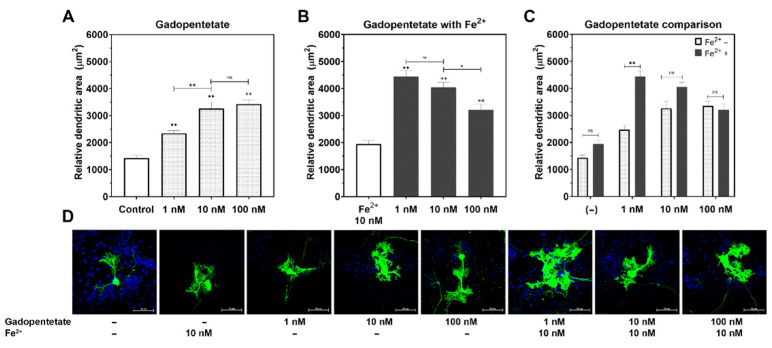
Changes in the relative dendritic area of the Purkinje cells post incubation with gadopentetate with/without Fe^2+^. (**A**) Gadopentetate significantly increased Purkinje cells’ dendrite arborization compared to the control group, particularly at 100 nM. (**B**) Fe^2+^ attenuated the effect of gadopentetate on the dendrite arborization. (**C**) The relative dendritic area of Fe^2+^ + gadopentetate-treated cells was significantly higher than for gadopentetate-treated cells. (**D**) Representative photomicrograph of gadopentetate-treated Purkinje cells. ** *p* < 0.01 and * *p* < 0.05 indicate a statistical significance by Tukey’s HSD post-hoc test compared to the control, unless indicated with a significance bar. ns: not significant.

**Figure 3:**
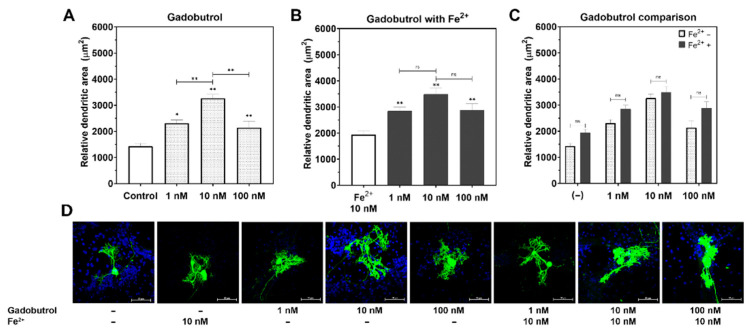
Changes in the relative dendritic area of the Purkinje cells post incubation with gadobutrol with/without Fe^2+^. (**A**) Representative photomicrograph of gadobutrol-treated Purkinje cells. (**B**) Incubation with gadobutrol significantly increased the Purkinje cell dendrite arborization compared to the control group, especially at 10 nM. (**C**) Fe^2+^ did not increase the effect of gadobutrol on dendrite arborization. (**D**) The relative dendritic area of Fe^2+^ + gadobutrol-treated cells was similar to that of the gadobutrol-treated cells. ** *p* < 0.01 and * *p* < 0.05 indicate a statistical significance by Tukey’s HSD post-hoc test compared to the control group, unless indicated with a significance bar. ns: not significant.

There were no significant differences in dendrite arborization between the Fe^2+^ and control groups. The dendrite arborization of the Purkinje cells increased in both the gadopentetate and gadobutrol groups (*p* < 0.01, respectively; Figure 2A and Figure 3A), compared to the control group. In the gadopentetate group, the greatest increase in dendrite arborization was observed when incubated with 100 nM gadopentetate (*p* < 0.01), followed by 10 nM (*p* < 0.01) and 1 nM (*p* < 0.01) (Figure 2A). Meanwhile, in the gadobutrol group, the highest increase was observed at a concentration of 10 nM (*p* < 0.01), followed by 1 nM (*p* < 0.01) and 100 nM (*p* < 0.01) (Figure 3A).

In the gadopentetate+Fe^2+^ group (Figure 2B), the greatest increase in dendrite arborization was observed when incubated with 1 nM gadopentetate (*p* < 0.01), followed by 10 nM (*p* < 0.01) and 100 nM (*p* < 0.01). In the gadobutrol+Fe^2+^ group (Figure 3B), an increase in dendrite arborization was observed at 10 nM (*p* < 0.01), followed by 1 nM (*p* < 0.01) and 100 nM, similar to the gadobutrol group.

There was a significant interaction between gadopentetate and Fe^2+^ (*p* < 0.01, η2 = 12.18, ANOVA). The incubation of 10 nM Fe with 1 nM gadopentetate significantly increased dendrite arborization, but not with 10 nM gadopentetate (*p* = 0.06) or 100 nM gadopentetate (*p* = 0.97; Figure 2C). In contrast, there was no interaction between gadobutrol and Fe^2+^ (*p* = 0.56, η2 = 1.21, ANOVA). The incubation of 10 nM Fe^2+^ did not increase dendrite arborization at any gadobutrol concentration (Figure 3C).

## 4. Discussion

Although both gadopentetate and gadobutrol were found to accelerate the thyroid hormone-induced dendrite arborization of the cerebellar Purkinje cells, only the effect of gadopentetate was augmented by Fe^2+^.

Thyroid hormones (TH) T3 and T4 are essential in the morphogenesis of Purkinje cells. Without TH, the growth and branching of Purkinje cells may be abnormal [22]. Molecular mechanisms coordinate dendrite arborization and ensure a functional neural network integrity [23]. One of the signaling molecules that takes part in the process of dendrite arborization is calcium/calmodulin-dependent protein kinase II (CaMKs). CaMK II is activated by intracellular calcium influx (Ca^2+^) and has autoinhibitory functions that inhibit and restrict dendrite growth [24,25]. Considering that Gd (107.8 pm) has an ionic radius similar to calcium (114 pm) [3,26], it can easily compete with Ca^2+^ in this site with a much higher affinity. Gd from GBCAs may impede the auto-inhibitory function of CaMK II. This may partially explain why Purkinje cell dendrite arborization by T4 was not coordinated properly when the neurons were incubated with gadopentetate or gadobutrol.

Although gadobutrol increased the dendrite arborization of cerebellar Purkinje cells compared to the control group, it was still noticeably lower when compared to gadopentetate. This suggested that chemical structure, especially thermodynamic stability, may be important in preventing Gd toxicity to neurons. In line with the study by Ariyani et al. [16], although gadodiamide and gadoterate were deposited in CV-1 cells, only gadodiamide altered the thyroid hormone receptor (TR)-mediated transcription, augmenting it at low doses but hampering cellular function at high doses. Both studies were reported in vitro and were performed using a mixed cell culture containing not only neurons but also astrocytes, oligodendrocytes, and microglia. Physiological iron levels are not uniform among the different cell types [27,28]. These cells have different densities and may affect the metabolism of ferrous iron and GBCAs in vitro.

**Figure 4:**
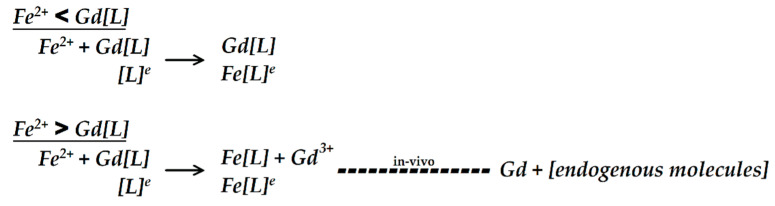
Proposed mechanism of how the concentration ratio between Fe^2+^ and chelated gadolinium (Gd[L]) contributes to transmetalation. When the Fe^2+^ concentration is lower than Gd[L] with excess ligand [L]^e^, Fe^2+^ will bind primarily with the [L]^e^ before competing with Gd[L]. However, when the Fe^2+^ concentration is higher than Gd[L], the [L]^e^ may not be sufficient for Fe^2+^ binding. Consequently, Fe^2+^ will compete with Gd[L], resulting in Gd^3+^ being released from its chelate (transmetalation). When transmetalation occurs in vivo, Gd^3+^ may bind with endogenous molecules such as phosphate or carbonate or may form a macromolecule complex.

One of the mechanisms for Gd retention in brain tissue is the transmetalation phenomenon, in which Gd is released from its chelate [29] due to the higher thermodynamic stability constant of Fe to DTPA (log Kcond: 23.4) when compared to Gd-DTPA (log Kcond: 18.4) [30]. Telgmann and colleagues [31] described that when gadopentetate was incubated with blood plasma in vitro for two hours, no compound of iron diethylenetriaminepentaacetic acid (Fe-DTPA) was detected, indicating the lack of transmetalation. Given that Magnevist^®^ solution contains an additional 0.2% (0.4 mg/mL; Table 1) of DTPA ligand [32,33], Fe^2+^ may primarily bind with this excess ligand instead of competing with chelated Gd, reducing transmetalation. We expected that the supplementation of Fe^2+^ in the medium would alter the GBCAs’ effect on thyroid hormone-induced dendrite arborization. Interestingly, our results suggested that the Fe^2+^-to-gadopentetate ratio may be important in augmenting the morphogenesis by the thyroid hormone, which in turn would affect the neuronal function. When the Fe^2+^ concentration was lower than the gadopentetate concentration (1:10), this effect may have been minimized. We suspected that Fe^2+^ binded with the excess ligand in the Magnevist^®^ solution (Figure 4). When the Fe^2+^ concentration was higher than gadopentetate (10:1), the 0.2% excess ligand might have been insufficient, and the remaining Fe^2+^ may have competed with the chelated Gd, increasing the release of Gd from the chelate. Under these conditions, transmetalation may occur more easily and affect the neurons, as indicated by the significant increase in the dendrite arborization of Purkinje cells.

The stability of gadobutrol, a macrocyclic contrast agent, supports this explanation. The higher affinity of Gd to dihydroxy-hydroxymethylpropyl-tetraazacyclododecane-triacetic acid (DO3A) would make it less likely for Fe^2+^ to trigger transmetalation. However, gadobutrol still affected arborization and its intensity at lower doses, especially 10 nM and 1 nM. This raises the question of whether intact contrast agents affect neurons through pathways similar to that of free Gd, despite being chelated. Gadopentetate is an ionic (higher osmolality) contrast agent, whereas gadobutrol is a non-ionic (lower osmolality) contrast agent. Ionic GBCAs have been shown to reduce calcium ions in vitro when compared with non-ionic GBCA [34], which may further explain why gadopentetate’s effect on dendrite arborization is much greater than gadobutrol.

Iron concentration needs to be balanced during brain development [17,35,36]. Iron overload may cause Purkinje cell loss and cellular damage via Fenton and Haber–Weiss reactions [37]. The Fe^2+^ concentration used in our study did not attenuate or suppress thyroid hormone-induced dendrite morphogenesis. However, the primary culture used in this study contained mixed types of cells, including astrocytes or glial cells. The physiological iron concentrations were different among the types of cells, being higher in glial cells than in neurons [28]. It is assumed that the major function of oligodendrocytes [38] requires high iron levels. Thus, we could not determine whether Fe^2+^ at a concentration of 10 nM would have affected Purkinje cells had the cells been cultured exclusively.

In addition to in vitro studies of Gd’s effect on neurons [8,16,39], animal studies have shown that Gd may affect the central nervous system and that Gd may be transferred to mice fetuses through the placenta, causing impaired brain development [40]. An in vivo study showed that residual Gd in the dentate nucleus and globus pallidus of the cerebellum was observed after only a single injection of gadopentetate, whereas multiple injections of gadobutrol were not associated with Gd retention in the cerebellum [41]. The phenomenon of in vivo Gd retention in the central nervous system, especially in the dentate nucleus of the cerebellum [42], sparks major concern about the risk of neurotoxicity, mainly in patients injected with linear GBCAs [43]. A notable absence of histological changes and neuropsychological deterioration related to GBCA injections was reported in a recent literature review [44]. Gd from GBCAs deposited in the brain tissue may not be toxic enough to cause histo-morphological changes or to manifest noticeable symptoms. The disparity among in vitro studies, animal studies, and the current clinical understanding must be considered before concluding whether retained Gd from GBCAs is toxic to humans. The choice of contrast agents in clinical practice should consider the GBCA’s safety profile for the patient’s benefit. Another important note is that Ca^2+^ is fundamental in mediating cellular excitability and is responsible for the biochemical regulation of the brain [45,46]. Ca^2+^ signaling involving voltage-gated calcium channels is also eminent in Purkinje cell development and mediates the transcription of neuronal morphogenesis, including dendrite arborization [47,48]. Because Gd is a strong Ca^2+^ antagonist [49], we expected that incubation with GBCAs and Fe^2+^ would suppress dendrite arborization. However, our study showed that both gadopentetate and gadobutrol increased dendrite arborization in Purkinje cells and showed a biphasic effect. Although the alteration of thyroid hormone receptors by GBCAs and the disruption of membrane receptor-mediated TH action have been proposed as underlying mechanisms [16], further studies are required to confirm this.

There were some limitations to this study. The Gd concentrations in the neurons were not quantified, and we could not confirm transmetalation using this study design. Although the addition of Fe^2+^ did not show a neurotoxicity effect, more detailed assays, such as a caspase 3 or tetrazolium assay, may be necessary to further elucidate the neurotoxicity of iron. Since the cell cultures contained mixed cells, there may have been interactions amongst cells, iron, and GBCAs. Furthermore, an in vivo study is required in order to extrapolate these results to clinical settings. In this in vitro study, the neurons were exposed directly to intact GBCAs, but in animal studies or a clinical setting, there are many variables before Gd from GBCAs can reach the brain (e.g., the blood–brain barrier, blood–cerebrospinal fluid barrier, and lymphatic system). Our study was not designed to determine whether GBCAs can enter the brain in an intact form or require binding with endogenous molecules to enter the brain.

## 5. Conclusions

In conclusion, the effect of GBCA on the thyroid hormone-induced cerebellar Purkinje cell arborization was dose-dependent. A higher dose of GBCA may significantly increase the dendrite arborization of Purkinje cells, and co-exposure with Fe^2+^ significantly increased the effect, most noticeably when the Fe^2+^ concentration was higher than the gadopentetate concentration. These findings suggested that the chelate thermodynamic stability and the concentration ratio between Fe^2+^ and GBCA may play important roles in triggering transmetalation, affecting the dendritogenesis of Purkinje cells in in vitro settings.

## Figures and Tables

**Table 1:** Characteristics of Magnevist^®^ and Gadovist^®^ [2,32,33].

	Gadopentetate Dimeglumine (Magnevist^®^)	Gadobutrol (Gadovist^®^)
Chemical Structures	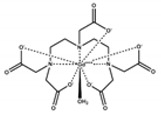 Gd-DTPA (Linear chelate)	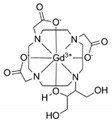 Gd-BT-DO3A (Macrocyclic chelate)
Molecular weight	938 g/mol	604.71 g/mol
Ionic charge	divalent ionic	non-ionic
Concentration	0.5 M	1.0 M
Osmolality	1960	1603
Excess ligand	0.4 mg/mL	-
log K_therm_	22.5	21.8
log K_cond_	18.4	14.7
Kinetic stability	Low	High

## Data Availability

The data presented in this study are openly available in [OSF data repository] at [https://osf.io/hs9g7/?view_only=13ec9c5a5cb143a2adda869e2a11fee5 (accessed on 12 October 2021)].
